# Memory CD8^+^ T Cell Protection From Viral Reinfection Depends on Interleukin-33 Alarmin Signals

**DOI:** 10.3389/fimmu.2019.01833

**Published:** 2019-08-07

**Authors:** Claudia Baumann, Anja Fröhlich, Tobias M. Brunner, Vivien Holecska, Daniel D. Pinschewer, Max Löhning

**Affiliations:** ^1^Experimental Immunology and Osteoarthritis Research, Department of Rheumatology and Clinical Immunology, Charité-Universitätsmedizin Berlin, Berlin, Germany; ^2^Pitzer Laboratory of Osteoarthritis Research, German Rheumatism Research Center (DRFZ), A Leibniz Institute, Berlin, Germany; ^3^Division of Experimental Virology, Department of Biomedicine, University of Basel, Basel, Switzerland

**Keywords:** IL-33, ST2, CD8^+^ T cells, alarmins, adaptive memory, virus infection

## Abstract

Memory CD8^+^ cytotoxic T lymphocytes (CTLs) can protect against viral reinfection. However, the signals driving rapid memory CTL reactivation have remained ill-defined. Viral infections can trigger the release of the alarmin interleukin-33 (IL-33) from non-hematopoietic cells. IL-33 signals through its unique receptor ST2 to promote primary effector expansion and activation of CTLs. Here, we show that the transcription factor STAT4 regulated the expression of ST2 on CTLs *in vitro* and *in vivo* in primary infections with lymphocytic choriomeningitis virus (LCMV). In the primary antiviral response, IL-33 enhanced effector differentiation and antiviral cytokine production in a CTL-intrinsic manner. Further, using sequential adoptive transfers of LCMV-specific CD8^+^ T cells, we deciphered the IL-33 dependence of circulating memory CTLs at various stages of their development. IL-33 was found dispensable for the formation and maintenance of memory CTLs, and its absence during priming did not affect their recall response. However, in line with the CTL-boosting role of IL-33 in primary LCMV infections, circulating memory CTLs required IL-33 for efficient secondary expansion, enhanced effector functions, and virus control upon challenge infection. Thus, beyond their effector-promoting activity in primary immune reactions, innate alarmin signals also drive memory T cell recall responses, which has implications for immunity to recurrent diseases.

## Introduction

An efficient activation and differentiation of effector CD8^+^ cytotoxic T lymphocytes (CTLs) is critical for the control of many viral infections. Once the infection is cleared, most effector cells (~95%) undergo apoptosis, while a long-lived population of memory cells survives (1). Thereafter, those memory cells are maintained by cytokines that provide signals for survival and homeostatic proliferation, most prominently IL-7 and IL-15 (2, 3). Upon viral challenge, memory CTLs are superior to their naive precursors in controlling secondary infections. This feature is attributed to a higher number of antigen-specific cells and a more rapid acquisition of effector functions upon antigen reencounter (4). Naïve CD8^+^ T cells require costimulatory signals for antigen-specific antiviral responses, while CD8^+^ memory T cells, at least in certain settings, can be reactivated independently of costimulation (5–7). In addition, the demand for cytokines differs between primary infection and recall responses (8, 9). The dependency of memory T cells on alarmin signals such as IL-33 has not, however, been studied yet.

IL-33 is a member of the IL-1 cytokine family and is constitutively expressed by endothelial and epithelial cells as well as by fibroblastic reticular cells in secondary lymphoid organs (10–12). Upon necrotic cell death, IL-33 is released, acting as an early damage signal, an alarmin, which can be sensed by several innate and adaptive immune cells (13–15). During infection with lymphocytic choriomeningitis virus (LCMV), splenic IL-33 mRNA levels increase. IL-33 signals through its receptor ST2, also known as T1 or IL-1 receptor-like-1 (IL-1RL1), on effector CTLs, to enhance their activation and antiviral functionality (16). So far, the role of IL-33 in T cell responses was mainly studied during primary effector activation. To address its function during memory formation, maintenance, and recall responses, we performed adoptive transfers of LCMV-specific CTLs into either IL-33-deficient or wild-type (WT) hosts, followed by LCMV infection, secondary crisscross T-cell transfer, and reinfection. This allowed us to assess the fate and challenge performance of memory CTLs when deprived of IL-33 at various stages of their development. We found that IL-33 is not required for the formation and maintenance of memory CTLs. However, it is critical for memory CTL re-expansion, efficient effector differentiation, and viral control during secondary infection. This finding emphasizes an unexpected role for alarmins as key drivers of protection by memory CD8^+^ T cell populations.

## Materials and Methods

### Mice

C57BL/6 (wild type, WT), *Stat4*^−/−^ (17), *Tbx21*^−/−^ (18), *Ifnar1*^−/−^ (19), *Il12p40*^−/−^ (20), *Ifngr1*^−/−^ (21), *Il1rl1*^−/−^ (22), and *Stat1*^−/−^ (23) mice were all backcrossed to C57BL/6 background and were used for CD8^+^ T cell isolation and/or in infection experiments. Transgenic mice expressing the P14-TCR specific for LCMV-H-2D^b^ (24) and CD45.1 as congenic marker on a C57BL/6 background were used as organ donors for the isolation of LCMV-specific CD8^+^ T cells. CD45.2-expressing C57BL/6 and *Il33*^−/−^ (25) mice were used as recipients in adoptive cell transfer experiments. *In vivo* experiments were performed with male and female mice at the age of 8–24 weeks. For adoptive T cell transfer experiments, T cells from male or female donor mice were transferred into male recipients to avoid rejection. When female recipients were used, donor T cells were derived from female mice. Animal protocols were performed in accordance with the German law for animal protection and the institutional guidelines of the Charité Berlin. All experiments were approved by the Landesamt für Gesundheit und Soziales in Berlin (LAGeSO, approval number G 0242/12).

### Virus Production and Virus Titer Determination

The LCMV-WE and LCMV-Clone 13 strains were propagated on L929 or BHK-21 cells, respectively. Virus stocks and organ samples were titrated by standard immunofocus assays on MC57G cells (26). In brief, MC57G cells were plated with organ homogenates or virus stock dilutions and subsequently overlaid with 2% methylcellulose. After 48 h of incubation at 37°C, the confluent monolayer of cells was fixed with 4% formaldehyde, permeabilized with 1% Triton X-100 (v/v) and stained with antibodies against LCMV nucleoprotein (VL-4). After a secondary staining step with peroxidase conjugated anti-rat IgG antibody (Jackson), foci were developed by 20 min incubation with OPD substrate (0.1 M Na_2_HPO_4_, 0.5 M citric acid, 0.03% H_2_O_2_, and 20 mg o-phenylenediamine dihydrochloride).

### Adoptive T Cell Transfer and Virus Infections

Naive CD45.1^+^ P14 CD8^+^ T cells were purified by magnetic cell sorting in a negative enrichment approach with biotin-labeled antibodies against CD4 (RM4-5), CD11b (M1/70), CD11c (HL3), CD25 (7D4), Gr-1 (RB6-8C5), and CD19 (1D3) in combination with anti-biotin microbeads (Miltenyi Biotec). For primary infections, 1.5 × 10^4^ purified P14 cells were transferred into either C57BL/6 or *Il33*^−/−^ mice, which were subsequently infected intravenously with a low dose (LD, 200 PFU) LCMV-WE in 200 μl MEM. P14 cells were analyzed at day 6.5 post infection. In the experiments presented in Figures 3–5, splenic CD45.1^+^ P14 CD8^+^ cells were isolated at day 16 after infection and FACS-sorted by depletion of CD4 (RM4-5), CD19 (1D3), and CD45.2 (104). For kinetic analysis without challenge infection, 1.5 × 10^6^ sorted CD45.1^+^ P14 CD8^+^ T cells were re-transferred into either C57BL/6 or *Il33*^−/−^ mice. For challenge infections, 1.5 × 10^4^ P14 cells were re-transferred into C57BL/6 or *Il33*^−/−^ mice. After 65 days, recipients were infected intravenously with a high dose (HD, 2 × 10^6^ PFU) LCMV-Clone 13 in 200 μl MEM and analyzed 6.5 days later.

### Primary T Cell Cultures

Naive CD8^+^ CD62L^hi^ CD44^−^ cells were sorted from pooled spleen and lymph node cells on a FACS Aria II. T cells were cultured in RPMI 1640+GlutaMax-I (Gibco) supplemented with 10% (v/v) FCS (Gibco), penicillin (100 U/ml; Gibco), streptomycin (100 μg/ml; Gibco), and ß-mercaptoethanol (50 ng/ml; Sigma) in the presence of APCs, antibodies against CD3 (145-2C11) and CD28 (37.51), both at 2.5 μg/ml, IL-2 (5 ng/ml), and anti-IL-4 (11B11, 10 μg/ml). In addition, IL-12 (10 ng/ml), IFN-γ (10 ng/ml), IFN-α and -β (each at 250 U/ml), or combinations thereof were added for effector differentiation. When indicated, plate-bound anti-CD3 (5 μg/ml) and anti-CD28 (3 μg/ml) were used without APCs. T cells were analyzed at day 5 of culture.

### Cell Isolation and Flow Cytometry

Single-cell suspensions of spleens were prepared by mechanical disruption. For cell isolation from livers, mechanical disruption was followed by digestion with Collagenase D (0.1 U/ml, Roche) for 30 min at 37°C. Then, lymphocytes were isolated using Histopaque-1083 and high-density centrifugation (400 g at 20°C for 20 min). When indicated*, ex vivo*-isolated lymphocytes and *in vitro*-differentiated CD8^+^ T cells were stained with antibodies against CD8 (53–6.7), CD45.1 (A20), CD62L (MEL-14), CD44 (IM7), KLRG1 (2F1), IL-18R (BG/IL18RA), CXCR3 (CXCR3-173), CD127 (A7R34), and PD-1 (J34). For flow-cytometric detection of cell surface ST2, splenocytes were stained with digoxigenin-coupled anti-mouse ST2 antibody (DJ8). For detection, a PE-coupled anti-digoxigenin Fab antibody (Roche) was used. To augment the PE signal, we performed two rounds of amplification using the PE FASER Kit (Miltenyi Biotec). LCMV-specific CD8 T cell response to the dominant glycoprotein-derived epitope GP33 was assessed by MHC class I tetramer staining as described previously (16). Samples were acquired on a FACS Canto II (BD), and analyzed with FlowJo (BD). Dead cells and doublets were excluded by a combination of forward scatter height and width gating and the usage of propidium iodide or a LIVE/DEAD fixable dye (BioLegend).

### Intracellular Cytokine and Transcription Factor Staining

For cytokine detection, *ex vivo* isolated cells were restimulated with GP33 peptide for 4 h with addition of brefeldin A (5 μg/ml; all from Sigma-Aldrich) at 30 min, followed by surface staining and fixation in 2% formaldehyde (Merck). Intracellular staining was performed in PBS/0.2% BSA containing 0.05% saponin (Sigma-Aldrich) with antibodies against IFN-γ (XMG1.2), IL-2 (JES6-5H4), and TNF-α (MPG-XT22). T-bet and Eomes protein amounts were analyzed using FoxP3 staining buffer set (eBioscience) according to the manufacturer's instructions. Briefly, cells were stained for surface marker expression and then fixed with 1x Fixation/Permeabilization buffer, followed by intracellular staining with antibodies against T-bet (4B10) and Eomes (Dan11mag) in 1x permeabilization buffer. Cells were washed in 1x permeabilization buffer and analyzed.

### Statistical Analysis

GraphPad Prism (v5.02 and v7) software was used for data analysis. Statistical significance was determined by unpaired two-tailed Student's *t*-test and Mann-Whitney *U* test as indicated in the figure legends. *P* = 0.01 to 0.05 was considered statistically significant (^*^), *p* = 0.001 to 0.01 as very significant (^**^), and *p* < 0.001 as extremely significant (^***^). n.s., not significant.

## Results

### ST2 Expression by CD8^+^ T Cells *in vitro* and *in vivo* Depends Largely on STAT4

**Figure 1:**
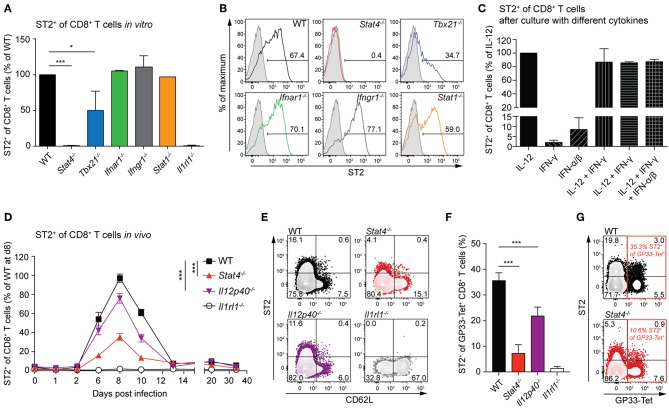
ST2 expression depends largely on STAT4 in CD8^+^ T cells *in vitro* and *in vivo*. **(A,B)** Naive CD8^+^ T cells from WT and *Stat4*^−/−^, *Tbx21*^−/−^, *Ifnar1*^−/−^, *Ifngr1*^−/−^, *Stat1*^−/−^, and *Il1rl1*^−/−^ mice were activated with soluble anti-CD3 and anti-CD28 plus APCs and differentiated into effector cells *in vitro* in the presence of IL-12 for 5 days. Mean + SD of ST2 frequency normalized to WT levels **(A)** in two to three independent cultures and representative histograms of ST2 expression **(B)** are shown (gray line: *Il1rl1*^−/−^ control staining). **(C)** Expression of ST2 on CD8^+^ T cells after *in vitro* differentiation with different combinations of cytokines and plate-bound antibodies against CD3 and CD28. Bars indicate the mean + SD of three independent cultures. **(D–G)** WT, *Stat4*^−/−^, *Il12p40*^−/−^, and *Il1rl1*^−/−^ mice were infected with a low dose (LD, 200 PFU) LCMV-WE. **(D)** Circulating CD8^+^ T cells were analyzed for ST2 expression. Symbols of the time course analyses represent the mean ± SEM values of two experiments (*n* = 5 per experiment). *P*-values were calculated for day 6, 8, and 10 post infection (two-way ANOVA with Bonferroni's *post-test*). **(E)** Representative contour plots of ST2 and CD62L expression of CD8^+^ T cells at day 8 after infection. **(F,G)** Quantification and representative contour plots of ST2^+^ GP33-tetramer^+^ CD8^+^ T cells on day 8 post infection. Bars indicate the mean + SD (*n* = 5). *P*-values were calculated with unpaired two-tailed Student's *t*-test **(A,C,F)**. ^*^P < 0.05 and ^***^P < 0.001.

IL-33 directly exerts its function on activated CD8^+^ T cells by signaling through its receptor ST2 (16, 27). We analyzed the ability of CD8^+^ T cells to express ST2 in the absence of various transcription factors and cytokine signals that are involved in CTL effector differentiation—STAT4, IL-12, T-bet, type-I and type-II interferons, and STAT1. To assess this *in vitro*, we analyzed ST2 frequencies of WT, *Stat4*^−/−^, *Tbx21*^−/−^, *Ifnar1*^−/−^, *Ifngr1*^−/−^, and *Stat1*^−/−^ CTLs, which were differentiated in the presence of IL-12. While WT, *Ifnar1*^−/−^, *Ifngr1*^−/−^, and *Stat1*^−/−^ CTLs showed comparable ST2 expression, it was reduced by half in *Tbx21*^−/−^ CTLs and absent in *Stat4*^−/−^ CTLs (Figures 1A,B). In line with this result, a lack of IL-12, which signals via STAT4, drastically reduced the frequencies of ST2 expression. IFN-γ or IFN-α/β without IL-12 was not sufficient to induce substantial ST2 expression, and adding them to IL-12 during differentiation did not further increase the frequencies of ST2^+^ cells (Figure 1C). Next, we addressed the effect of STAT4 and IL-12 signals on ST2 expression by CTLs *in vivo*. We infected WT, *STAT4*^−/−^, *Il12p40*^−/−^, and ST2-deficient *Il1rl1*^−/−^ control mice with 200 PFU LCMV-WE, an infection setting known to result in unimpaired virus clearance in all of the aforementioned gene-targeted mice (16, 28, 29). ST2 expression on circulating CTLs was transient and peaked at day 8 after infection, coinciding with the peak CTL response. *Stat4*^−/−^ CD8^+^ T cells exhibited weakest ST2 expression (Figures 1D,E). In conjunction with the observation that T cell-intrinsic STAT4 signals were vital for ST2 expression *in vitro* (cf. Figure 1A), it seems likely that impaired ST2 expression *in vivo* was also due to a T cell-intrinsic STAT4 deficiency. As effector frequencies (marked by CD62L down-regulation) varied between the different genotypes (Figure 1E), we used MHC class I tetramers to determine ST2 expression on effector CTLs that were specific for the LCMV glycoprotein-derived epitope GP33. Consistent with ST2 expression patterns in the total CD8^+^ T cell population, only a very small proportion of GP33-specific *Stat4*^−/−^ T cells displayed ST2 expression, and significantly reduced ST2 levels were also observed in CTLs from *Il12p40*^−/−^ mice (Figures 1F,G). These findings show that ST2 expression on CTLs depends largely on STAT4, both *in vitro* and *in vivo*.

### IL-33 Enhances CD8^+^ T Cell Activation and Differentiation During a Primary Viral Infection

**Figure 2:**
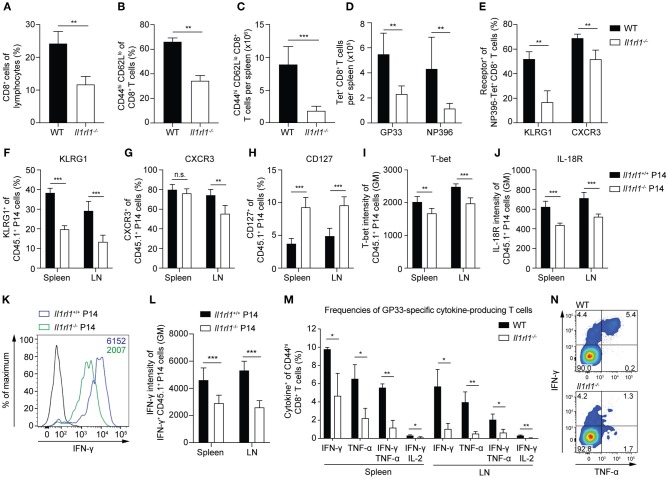
IL-33 enhances CD8^+^ T cell activation and differentiation during a primary viral infection. **(A–E)** WT and *Il1rl1*^−/−^ mice were infected with a low dose (LD, 200 PFU) LCMV-WE and spleens were analyzed at day 6.5 after infection (*n* = 5). **(A)** Frequency of CD8^+^ T cells among lymphocytes. Frequency **(B)** and absolute numbers **(C)** of splenic CD44^hi^ CD62L^lo^ effector CD8^+^ T cells. Total count of GP33-and NP396-tetramer^+^ CD8^+^ T cells per spleen **(D)** and frequency of KLRG1^+^ and CXCR3^+^ cells among NP396-tetramer^+^ CD8^+^ T cells **(E)**. **(F–L)** Naive *Il1rl1*^+/+^ and *Il1rl1*^−/−^ P14 cells were transferred into WT recipients. Recipients were subsequently infected with a low dose (LD, 200 PFU) LCMV-WE. P14 cells from spleen and lymph nodes were analyzed at day 6.5 after infection (*n* = 5–6). Frequencies of KLRG1^+^ **(F)**, CXCR3^+^ **(G)**, and CD127^+^ **(H)** cells among CD45.1^+^ P14 cells. Expression levels of T-bet **(I)** and IL-18R **(J)** of CD45.1^+^ P14 T cells plotted as geometric mean (GM) of fluorescence intensity. Representative histogram **(K)** and quantification **(L)** of IFN-γ production by *Il1rl1*^+/+^ and *Il1rl1*^−/−^ P14 cells after restimulation with GP33 peptide (black histogram: unstimulated *Il1rl1*^+/+^ P14 cells). **(M,N)** WT and *Il1rl1*^−/−^ mice were infected with a low dose (LD, 200 PFU) LCMV-WE. Spleen and lymph nodes were analyzed after GP33 peptide restimulation at day 6.5 post infection. **(M)** Frequencies of cytokine-producing GP33-specific CD44^hi^ CD8^+^ T cells and a representative FACS plot of IFN-γ and TNF-α expression **(N)** by CD44^hi^ CD8^+^ T cells. Bars indicate the mean + SD (*n* = 3). *P*-values were calculated with unpaired two-tailed Student's *t*-test. ^*^P < 0.05, ^**^P < 0.01, and ^***^P < 0.001.

To characterize the activation and differentiation states of CD8^+^ T cells primed without IL-33 signals, we infected WT and *Il1rl1*^−/−^ mice with LCMV and analyzed their CTLs at day 6.5 after infection. Already at this early time point, the absence of IL-33 signaling impaired the expansion of the splenic CD8^+^ T cell pool (Figure 2A) and consequently reduced the frequency and absolute numbers of CD44^high^ CD62L^low^ CTLs (Figures 2B,C). Analysis of LCMV-specific T cells by tetramer staining showed reduced numbers of GP33- and NP396-specific CTLs in ST2-deficient mice (Figure 2D). Moreover, a lack of IL-33 signaling reduced the frequency of KLRG1- and CXCR3-expressing LCMV-specific CD8^+^ T cells, which reflects reduced effector differentiation (Figure 2E). To demonstrate that reduced CTL activation was due to direct sensing of IL-33 by CD8^+^ T cells and not due to indirect effects on other cell types, we transferred *Il1rl1*^−/−^ P14 and *Il1rl1*^+/+^ P14 cells into WT mice and subsequently infected the recipients with LCMV. At day 6.5 post infection, ST2-deficient P14 cells isolated from spleen and lymph nodes showed a reduced expression of KLRG1 (Figure 2F). CXCR3 expression was significantly reduced on *Il1rl1*^−/−^ P14 cells isolated from lymph nodes (Figure 2G), and CD127 expression was increased on these cells in spleen and lymph nodes (Figure 2H). As both KLRG1 and CXCR3 expression are controlled by the transcription factor T-bet (30, 31) we assessed its expression level in the transferred P14 cells. The absence of IL-33 signals significantly reduced T-bet levels (Figure 2I). Likewise, IL-18R expression intensity was decreased (Figure 2J). Reduced CTL activation in the absence of ST2 was also reflected by a lower expression intensity of IFN-γ per cell (Figures 2K,L). In addition, we found reduced frequencies of virus-specific, IFN-γ-producing CTLs, expressing IFN-γ alone or in combination with TNF-α and IL-2 in *Il1rl1*^−/−^ mice at 6.5 days post LCMV infection (Figures 2M,N). Taken together, our data show that a lack of IL-33 signals leads to lower T-bet and type-I cytokine expression amounts in CTLs and severely impairs their activation, expansion, and effector differentiation during a primary antiviral response.

### IL-33 Is Dispensable for CD8^+^ T Cell Memory Formation and Maintenance

**Figure 3:**
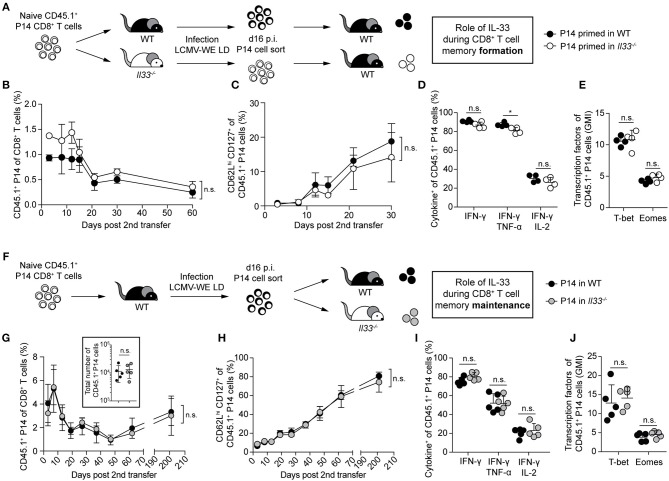
IL-33 is dispensable for CD8^+^ T cell memory formation and maintenance. **(A)** Schematic experimental layout to assess IL-33 signals during memory formation in **(B–E)**. **(B)** Frequency of P14 cells in the blood of WT recipients at the indicated time points after secondary transfer. **(C)** Frequency of CD62L^hi^ CD127^+^ cells within the P14 cell subset. Symbols of the time course analyses represent mean ± SD (*n* = 4) of one representative experiment out of three performed. **(D)** After 65 days, recipients were sacrificed. Cytokine production of splenic P14 cells after GP33 restimulation. **(E)** Expression levels of T-bet and Eomes by splenic P14 cells (geometric mean index, normalized to isotype). **(F)** Schematic experimental layout to assess IL-33 signals during memory maintenance in **(G–J)**. **(G)** Frequency of transferred P14 cells in the blood of secondary recipients and absolute numbers of P14 cells in the spleen at day 65 post second transfer (insert panel). **(H)** Frequency of CD62L^hi^ CD127^+^ cells within the P14 cell subset. Symbols of the time course analyses represent mean ± SD (*n* = 5) of one out of two experiments with similar results. **(I)** After 65 days, recipients were sacrificed in some experiments. Cytokine production by splenic P14 cells after GP33 peptide restimulation. **(J)** Expression levels of T-bet and Eomes by P14 cells (geometric mean index, normalized to isotype). Symbols represent single mice, the bars the mean + SD. *P*-values were calculated with unpaired two-tailed Student's *t*-test [starting-point-normalized Area under the curve (AUC) in **(B,G)**, AUC in **(C,H)**]. ^*^P < 0.05.

To assess whether a lack of IL-33 during priming affects the formation of memory CTLs, we adoptively transferred LCMV-specific CD8^+^ T cells (P14 cells) either into WT or IL-33-deficient primary recipient mice, followed by infection with 200 PFU LCMV. After 16 days, when clearance of such a low-dose LCMV infection can be assumed (16), the progeny of these CTLs were isolated again and equal numbers were re-transferred into naive WT secondary recipients (Figure 3A). Time course analyses from blood revealed that both CTL populations were maintained similarly (Figure 3B) and upregulated CD127 and CD62L comparably over time (Figure 3C). When restimulated with peptide on day 65 after second transfer, the production of IFN-γ, either alone or in combination with TNF-α and IL-2, was comparable in both CTL populations (Figure 3D). Furthermore, the expression levels of T-bet and Eomes by splenic P14 cells were virtually identical (Figure 3E). These data indicate that the lack of IL-33 during the priming phase does neither impair the memory differentiation of effector CTLs nor their maintenance over time.

Yet, we wondered whether withdrawal of IL-33 signals during the memory phase impairs CTL maintenance. For this, P14 cells were transferred into primary WT hosts, primed by LCMV infection, and then re-transferred into WT or *Il33*^−/−^ secondary recipients (Figure 3F). Again, the maintenance of both CTL populations (Figure 3G) and their CD62L and CD127 profiles (Figure 3H) were indistinguishable for >200 days. Also, their cytokine production upon peptide restimulation (Figure 3I) and the expression of T-bet and Eomes (Figure 3J) were comparable. This shows that IL-33 is neither required for CTL memory formation nor maintenance.

### Lack of IL-33 During Priming Does Not Impair Memory CTL Protection Against Viral Reinfection

**Figure 4:**
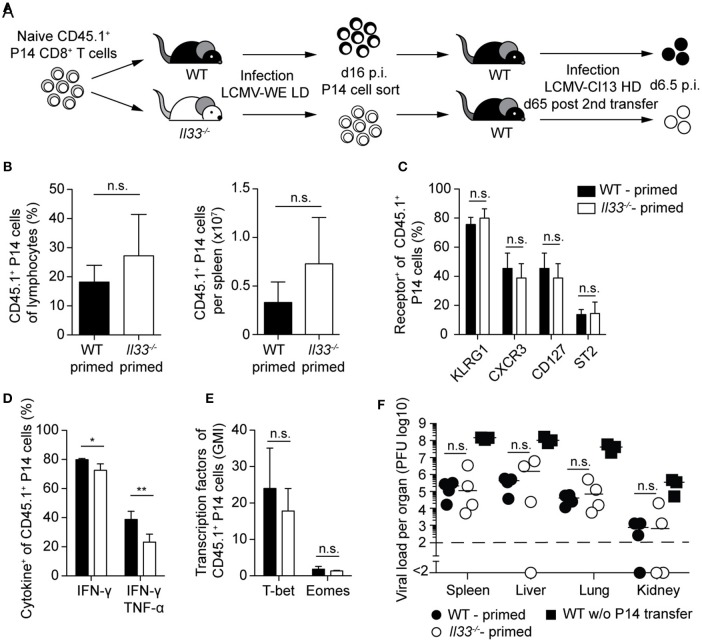
Lack of IL-33 during priming does not impair protection by memory CTLs upon viral reinfection. **(A)** Schematic experimental layout to assess the impact of IL-33 signals during priming on the outcome of a challenge infection. **(B)** Frequency and total P14 cell numbers in the spleen. **(C)** Expression of cell-surface markers by splenic P14 cells. **(D)** Cytokine production by P14 cells in the spleen after GP33 restimulation. **(E)** Expression levels of T-bet and Eomes by P14 cells (geometric mean index, normalized to isotype). **(F)** Viral titers in the organs indicated. Bar graphs in **(B–E)** show the mean + SD (*n* = 4). Symbols in F represent single mice, the bars the median. Each experiment was performed two to three times. *P*-values were calculated with unpaired two-tailed Student's *t*-test **(B–E)** and Mann-Whitney *U* test **(F)**. ^*^P < 0.05 and ^**^P < 0.01.

Next, we asked whether a lack of IL-33 signals during CTL priming, causing reduced peak expansion and effector differentiation in the primary response (cf. Figure 2), would translate into durable cell-intrinsic defects. These effects could then manifest in a defective secondary antiviral response even when tested in an IL-33-competent environment (Figure 4A). When adoptively transferred into WT secondary hosts, memory P14 cells derived from WT or *Il33*^−/−^ primary recipients expanded to similar frequencies and total numbers after LCMV challenge infection (Figure 4B). Moreover, P14 cells from both groups exhibited comparable expression of the cell surface receptors KLRG1, CXCR3, CD127, and ST2 (Figure 4C). While the formerly IL-33-deprived P14 cells produced slightly less IFN-γ and TNF-α after peptide restimulation (Figure 4D), the expression levels of T-bet and Eomes were comparable (Figure 4E). Functionally, the minor differences in cytokine production between the two P14 CTL populations did not translate into differential viral clearance. When compared to mice without P14 cell transfer, viral titers in spleen, liver, lung, and kidney were reduced to a similar extent irrespective of the source of memory P14 cells transferred (Figure 4F). In summary, memory CTLs emerging from an IL-33-deprived environment were fully functional and protective, and displayed a largely normal phenotype upon secondary infection.

### Memory CTLs Require IL-33 for a Protective Antiviral Recall Response

**Figure 5:**
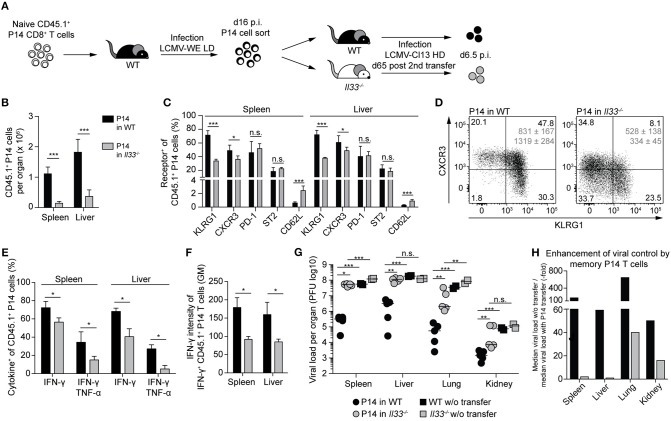
Memory CTLs require IL-33 for a protective antiviral recall response. **(A)** Schematic experimental layout to study the role of IL-33 during a recall response. **(B)** Total numbers of P14 cells in spleen and liver. **(C)** Expression of surface markers by P14 cells from spleen and liver. **(D)** Representative FACS plots of CXCR3 and KLRG1 expression by splenic P14 cells. Average geometric means of CXCR3 (top) and KLRG1 (bottom) ± SD are depicted in gray. **(E)** Cytokine production by P14 cells from spleen and liver after GP33 restimulation. **(F)** Expression levels of IFN-γ by IFN-γ^+^ P14 cells in spleen and liver. **(G)** Viral titers in the organs indicated. Symbols in **(H)** represent single mice, the bars the median. **(H)** Factors of enhanced viral clearance by memory P14 cells were calculated by dividing the median viral load of mice without cell transfer by the median viral load of mice with P14 cell transfer. Bar graphs in **(B–G)** indicate the mean + SD (*n* = 5). Each experiment was performed twice. *P*-values were calculated with unpaired two-tailed Student's *t*-test **(B,C,E,F)** and Mann-Whitney *U* test **(G)**. ^*^P < 0.05, ^**^P < 0.01, and ^***^P < 0.001.

During primary infection, IL-33 enhances the response of naive CD8^+^ T cells (cf. Figure 2) and is necessary for virus control (16). Thus, we wondered whether the antiviral recall response of an established CTL memory population still depends on IL-33 signals. To address this, adoptively transferred naive P14 cells were primed with LCMV in WT primary recipients. The resulting P14 memory cells were then adoptively re-transferred into WT or *Il33*^−/−^ secondary hosts and were allowed to rest for 65 days to ensure, to the extent possible, their memory differentiation prior to LCMV re-challenge infection (Figure 5A). The expansion of P14 memory cells was >10-fold reduced when the cells were challenged in IL-33-deficient recipients as opposed to WT hosts (Figure 5B). Moreover, P14 cells in *Il33*^−/−^ mice showed a less activated effector phenotype, as evident in lower KLRG1 and CXCR3 expression and higher expression of CD62L (Figures 5C,D). In line with this, the expression of IFN-γ and TNF-α upon peptide restimulation was impaired in *Il33*^−/−^ hosts (Figure 5E). Beyond the lower frequency of cytokine^+^ CTLs, the average amounts of IFN-γ produced by IFN-γ-positive cells were also reduced (Figure 5F). As compared to their naive counterparts, memory CTLs are more efficient in controlling a challenge infection (4). Indeed, when we compared WT mice with and without P14 cell transfer, we found that P14 memory cells potently suppressed viral replication (Figure 5G). In contrast, P14 memory cells in *Il33*^−/−^ hosts contributed little, if any, to viral control (Figure 5G). While memory P14 cells reduced viral loads in WT recipient spleens 170-fold, no more than a two-fold reduction resulted when the same cells were transferred to *Il33*^−/−^ recipients. This reflected a drastic improvement of memory CTL-mediated virus control in IL-33-competent mice (Figure 5H). Thus, memory CTLs need IL-33 in an antiviral recall response for efficient re-expansion, effector differentiation, and protection.

## Discussion

Both naive and memory CD8^+^ T cells require cytokines as co-factors for their activation, yet their specific demands differ (8, 9, 32). The alarmin IL-33 was shown to enhance primary CTL responses, but the molecular regulation of IL-33 receptor expression and the impact of IL-33 signals on an antiviral recall response of memory CTLs remained ill-defined. In our study, we showed that expression of the IL-33 receptor ST2 on CTLs largely depends on STAT4 and to some extent also on IL-12. While IL-33 signals enhance antiviral CTL effector differentiation in a primary response, the formation and maintenance of memory CTLs were independent of IL-33. Conversely, memory CTLs required IL-33 for a protective antiviral recall response.

Activated STAT4 plays a critical role in chromatin modification and gene expression (33) and is increased in CTLs responding to an LCMV infection (34). In CD4^+^ T cells, STAT4 induces ST2 expression together with T-bet (35, 36), possibly due to the ability of STAT4 and/or T-bet to open the ST2-encoding gene locus *Il1rl1* in the context of T cell effector differentiation (33). This is in line with our findings of STAT4-dependent ST2 expression in CTLs. It remains unclear whether the STAT4 effect is mediated directly or indirectly, e.g., by promoting T-bet expression. STAT4 phosphorylation is prominently mediated by IL-12 (37), but also by type-I IFNs, which upon LCMV infection are released in much higher amounts than IL-12 (38). Thus, we assume that type-I IFN-triggered STAT4 activation can partially compensate for the lack of IL-12, explaining why defective ST2 expression is more prominent in *Stat4*^−/−^ CTLs than in those of *Il12p40*^−/−^ mice. Yet, STAT4 deficiency did not completely abrogate ST2 expression *in vivo*. CD8^+^ T cells, albeit at a low level, express the transcription factor GATA-3, which has been shown to regulate ST2 expression in Th2 cells, and its absence compromises the expansion of virus-specific CD8^+^ T cells in an acute LCMV infection (39). It is conceivable that in the absence of STAT4, residual ST2 expression might be driven by GATA-3.

While primary CTL responses are enhanced by IL-33, formation of circulating memory CD8^+^ T cell was fully supported in a IL-33-deficient environment. Likewise, the maintenance of memory CTLs is independent of IL-33 signals. This is consistent with the transient and activation-dependent nature of ST2 expression on CTLs. Absence of ST2 expression on circulating CD8^+^ T cells during memory formation and maintenance might help preventing the undesired activation of CTLs in the absence of cognate antigen. The possibility to selectively act on activated CD8^+^ T cells in a very short time frame during the effector phase distinguishes IL-33 from other cytokines such as IL-7 and IL-15, which provide signals for memory CTL homeostatic proliferation and population maintenance (2, 3). It has been reported that IL-2 plays an important role during primary infection in programming the development of memory CTLs to ensure full secondary expansion upon challenge (8). IL-33 and IL-2 exhibit functional similarities in their capacity to enhance primary CTL responses. Yet, the absence of IL-33 signals during CTL priming did not phenocopy the IL-2-deprived “helpless” memory CTL, and a defective primary effector T cell differentiation did not translate into a permanent cell-intrinsic impairment. On the contrary, CTL priming in the absence of IL-33 still allowed for protective memory CTL recall responses provided the cells were challenged in an IL-33-sufficient environment.

During a recall response, memory CTLs re-expressed ST2 and required IL-33 for efficient reactivation and virus control. Notably, ST2 expression levels were identical between CD8^+^ memory cells reactivated in *Il33*^−/−^ or WT mice, indicating that ST2 expression in antiviral CTLs is independent of IL-33 signals. In memory Th2 cells and regulatory T cells, ST2 expression depends on the transcription factor GATA-3, and IL-33 signaling can induce GATA-3 phosphorylation (40–42). This suggests a positive feedback loop, in which IL-33 binding to its receptor ST2 elicits GATA-3 phosphorylation, which in turn can lead to nuclear translocation of GATA-3 and enhancement of ST2 expression (15). This mechanism could explain the increase of ST2 expression by IL-33 in memory Th2 cells (42). In contrast, our results show that in CD8^+^ T cells, ST2 expression is to a large extent dependent on the transcription factor STAT4. So far, there is no evidence that IL-33 signaling can modulate STAT4 activity, implying a lack of positive feedback mechanisms affecting ST2 expression in CD8^+^ T cells. Similar to IL-33 effects in the primary response, the selective expression of ST2 on activated CD8^+^ memory T cells may facilitate the preferential secondary expansion of antigen-specific circulating effector cells. Additionally, IL-33 enhances effector differentiation and boosts cytokine production at a single cell level, thus acting in a multi-tiered process to achieve efficient virus control. This finding is in line with the notion of IL-33 as an alarmin—an early damage signal that activates innate and adaptive immune cells (43, 44). It is surprising though that also memory cells, which have an advanced differentiation state and respond faster than naive cells (45, 46), require IL-33 to unleash their full protective potential. Thereby, IL-33 can fine tune both primary and secondary immune responses based on the magnitude of its cell damage-associated release.

Our experimental setup of adoptive CD8^+^ T cell transfers between WT and IL-33-deficient mice (and vice versa) allowed us to deprive CTLs of IL-33 signals selectively during different stages of their differentiation. In contrast to a transfer of ST2-deficient P14 cells, this approach enabled us to address the relevance of IL-33 signals during memory formation, maintenance, and re-challenge without compromising the primary activation of these LCMV-specific CD8^+^ T cells. However, this experimental strategy has its limitations as it levels out numerical differences between CTLs primed in the presence or absence of IL-33. Such differences in cell numbers, albeit minor (16), might still affect the outcome of a secondary response. This was shown in a vaccination study utilizing IL-33 DNA constructs to increase the size of the CTL memory pool (47). Moreover, the requirement for IL-33 signals might differ between circulating and tissue-resident memory T cells (T_RM_). It was shown recently that IL-33 signals are essential for inflationary expansion of CD8^+^ T_RM_ in a chronic cytomegalovirus infection (48). Our experimental approach of transferring equal numbers of P14 CD8^+^ T cells into IL-33-deficient or -sufficient recipient mice was chosen to study the role of IL-33 in a cell-intrinsic manner. Still, the theoretical possibility remains that ST2-expressing cells other than CD8^+^ T cells contribute to the phenotypes observed or that the lack of IL-33 indirectly affects the antiviral immune response by altering various other immune compartments under homeostatic conditions. In fact, the lack of IL-33 has been shown to dampen the local activation of type 2 innate lymphoid cells (ILC2s), already in the steady state (49–51). It therefore is conceivable that IL-33 may affect the homeostasis of conventional ILC1s or natural killer cells, which can contribute to antiviral immune responses (52, 53). Nevertheless, we consider it highly unlikely that such alterations would have substantial effects on the parameters studied here. First, it is well established that acute viral clearance in the LCMV challenge model is virtually exclusively mediated by CD8^+^ T cells (54). Second, our previous work using both adoptive T cell transfer and bone-marrow chimera experiments established that IL-33 augments antiviral responses by acting directly on ST2-expressing CTLs (16). In conclusion, our findings reveal an unexpected role of alarmins as important co-factors in antiviral CTL recall responses. Hence, the manipulation of alarmin signals such as IL-33 may offer opportunities for improved therapies of recurrent viral diseases.

## Data Availability

All datasets generated for this study are included in the manuscript and/or the supplementary files.

## Ethics Statement

Animal protocols were performed in accordance with the German law for animal protection and the institutional guidelines of the Charité Berlin. All experiments were approved by the Landesamt für Gesundheit und Soziales in Berlin (LAGeSO, approval number G 0242/12).

## Author Contributions

CB, AF, TB, DP, and ML designed the research. CB, AF, TB, and VH performed the research. CB, AF, and TB analyzed the data. CB and ML wrote the paper.

### Conflict of Interest Statement

The authors declare that the research was conducted in the absence of any commercial or financial relationships that could be construed as a potential conflict of interest.
